# Dry Beriberi During Pregnancy After Hyperemesis Gravidarum: A Case Report of Axonal Sensorimotor Polyneuropathy

**DOI:** 10.7759/cureus.109518

**Published:** 2026-05-23

**Authors:** Hajar Yazough, Hajar Menay, Ahmed Amine El Oumri

**Affiliations:** 1 Department of Physical Medicine and Rehabilitation, Mohammed VI University Hospital of Oujda, Faculty of Medicine, Mohammed First University, Oujda, MAR

**Keywords:** dry beriberi, hyperemesis gravidarum, pregnancy, rehabilitation, sensorimotor axonal polyneuropathy, thiamine deficiency

## Abstract

Thiamine (vitamin B1) deficiency is a preventable complication of prolonged hyperemesis gravidarum, and the resulting malnutrition may lead to disabling peripheral neuropathy. We report the case of a 25-year-old primigravida who presented at 20 weeks of gestation with rapidly progressive distal weakness and glove-and-stocking paresthesias. Electroneuromyogram testing revealed an axonal sensorimotor polyneuropathy, and the serum thiamine level was low, supporting the diagnosis of thiamine deficiency-related neuropathy (dry beriberi). High-dose intravenous thiamine resulted in partial neurological improvement. A structured multidisciplinary inpatient rehabilitation program led to major functional gains, with the patient progressing from wheelchair dependence to independent ambulation within two months. This case highlights the importance of early recognition of thiamine deficiency in pregnancy and the added value of a structured multidisciplinary physical medicine and rehabilitation program, alongside prompt thiamine replacement, in restoring safe ambulation and functional independence during pregnancy.

## Introduction

Thiamine (vitamin B1) is an essential water-soluble vitamin involved in carbohydrate metabolism and neuronal function, and deficiency can lead to neurological manifestations, including Wernicke encephalopathy, cardiomyopathy, and peripheral neuropathy. Pregnancy is a period of increased metabolic demand during which prolonged vomiting, inadequate dietary intake, and malnutrition may precipitate thiamine deficiency, particularly in the setting of hyperemesis gravidarum [1].

Thiamine deficiency-related neuropathy is potentially reversible when recognized and treated promptly; however, delayed diagnosis may result in severe functional impairment and prolonged disability [2]. Peripheral neuropathies during pregnancy also pose diagnostic and therapeutic challenges, requiring careful attention to maternal recovery, fetal safety, and prevention of complications such as falls and loss of autonomy [3].

In addition to prompt thiamine replacement, functional recovery in thiamine deficiency-related neuropathy may be enhanced by targeted physical medicine and rehabilitation (PMR) strategies. This case is noteworthy because it illustrates a particularly severe axonal sensorimotor polyneuropathy due to thiamine deficiency in the context of prolonged hyperemesis gravidarum and provides a detailed account of the structured rehabilitation program and functional recovery during pregnancy.

## Case presentation

A 25-year-old primigravida with no significant past medical history presented at 20 weeks' gestation with a subacute history of rapidly progressive bilateral limb weakness. Two months prior to admission, she had experienced intractable hyperemesis gravidarum complicated by prolonged malnutrition and significant weight loss. One month prior to admission, she developed an insidious sensation of heaviness in the lower limbs, followed by progressively worsening bilateral lower-limb weakness, gait disturbance, and recurrent falls. Concurrently, she reported distal paresthesias in a glove-and-stocking distribution involving both the hands and feet. Over the subsequent weeks, the weakness progressed to a complete inability to walk and subsequently involved the distal upper limbs. Notably, there was no altered mental status, ophthalmoplegia, seizures, or sphincter dysfunction, and the patient remained afebrile throughout.

On physical examination, the patient was asthenic but alert and fully oriented in time, place, and person, with stable hemodynamic and respiratory parameters. Neurological examination revealed bilateral, symmetrical muscle weakness graded 3/5 distally and 4/5 proximally on the Medical Research Council (MRC) scale, with focal amyotrophy of the left hamstrings. Distal glove-and-stocking paresthesias were elicited on sensory testing, and deep tendon reflexes were globally diminished. Standing and independent ambulation were impossible without physical assistance. The remainder of the systemic examination was unremarkable.

Brain MRI, including axial FLAIR and sagittal T1-weighted sequences, revealed no intracranial signal abnormalities and no evidence of Wernicke encephalopathy (Figure 1).

**Figure 1:**
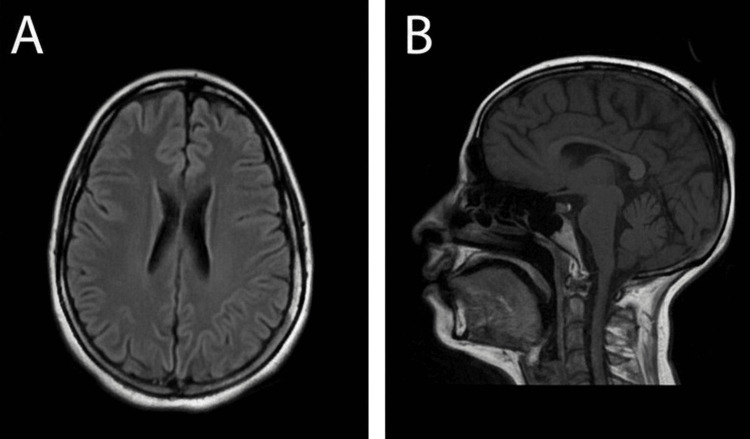
Brain MRI showing no intracranial abnormality: (A) axial FLAIR image and (B) sagittal T1-weighted image MRI: magnetic resonance imaging, FLAIR: fluid-attenuated inversion recovery

Laboratory investigations demonstrated a markedly low serum thiamine level of 9 µg/L (reference range: 28-85 µg/L), consistent with severe deficiency. Supplementary nutritional and metabolic workup revealed: serum thiamine at 320 pg/mL (reference range: 200-900 pg/mL), serum folate at 8.5 ng/mL (reference range: 3-17 ng/mL), and serum albumin at 28 g/L (below the lower limit of normal, consistent with protein-energy malnutrition). Cerebrospinal fluid analysis showed protein at 0.35 g/L, glucose at 3.2 mmol/L, and a white blood cell count of 2/mm³, with no albuminocytologic dissociation. Routine investigations, including complete blood count, C-reactive protein, serum electrolytes, liver and renal function tests, and coagulation studies, were all within normal limits. Serologic testing for HIV, hepatitis B, and syphilis returned negative results.

Electrophysiological evaluation of all four limbs revealed findings consistent with active axonal sensorimotor polyneuropathy with predominant distal involvement. Motor nerve conduction studies demonstrated markedly reduced compound muscle action potential (CMAP) amplitudes in the common peroneal nerve (distal CMAP: 2.9 mV; amplitude reduction >50% relative to the lower limit of normal) with preserved conduction velocities across all segments (41.7-48.1 m/s) and no evidence of conduction block; tibial motor responses were relatively spared. In the upper limbs, median motor responses were largely preserved, while the left ulnar nerve showed an absent distal CMAP at the wrist, consistent with severe distal axonal loss. Sensory nerve conduction studies revealed uniformly low-amplitude sensory nerve action potentials (SNAPs): sural 1.4 µV and median 4.0 µV, with mildly reduced conduction velocities, consistent with predominant distal sensory axonal involvement. F-wave latencies were within normal limits across all tested nerves, thereby excluding a demyelinating process. Distal motor latencies did not exceed 125% of the upper limit of normal. These findings collectively fulfilled established electrophysiological criteria for a length-dependent axonal sensorimotor polyneuropathy. Selected nerve conduction parameters are presented in Table 1, and representative electrophysiological traces are illustrated in Figure 2, showing reduced common peroneal CMAP amplitudes without conduction block and a low-amplitude left sural SNAP, consistent with distal axonal sensorimotor involvement.

**Table 1:** Selected motor and sensory nerve conduction study parameters Findings consistent with a length-dependent axonal sensorimotor polyneuropathy with predominant distal involvement. R: right, L: left, CMAP: compound muscle action potential, SNAP: sensory nerve action potential, NR: no response, CV: conduction velocity

Nerve (side)	Type	Site	Distance (mm)	Latency (ms)	Amplitude (mV/μV)	CV (m/s)	Interpretation
Common peroneal nerve (R)	Motor	Ankle	80	4.5	2.9 mV	-	Reduced distal CMAP amplitude
Common peroneal nerve (R)	Motor	Fibular head	350	12.9	2.8 mV	41.7	No conduction block; conduction velocity relatively preserved
Common peroneal nerve (R)	Motor	Popliteal fossa	100	15.0	2.7 mV	48.1	No conduction block; conduction velocity relatively preserved
Sural nerve (L)	Sensory	Distal leg (lower third)	110	2.62-3.04	1.4 μV	42.0	Low-amplitude SNAP; mildly reduced conduction velocity
Median nerve (L)	Sensory	Palm	80	2.35	4.0 μV	34.0	Low-amplitude SNAP; mildly reduced conduction velocity
Ulnar nerve (L)	Motor	Wrist	-	NR	0 mV	-	Absent distal CMAP, consistent with severe distal axonal loss

**Figure 2:**
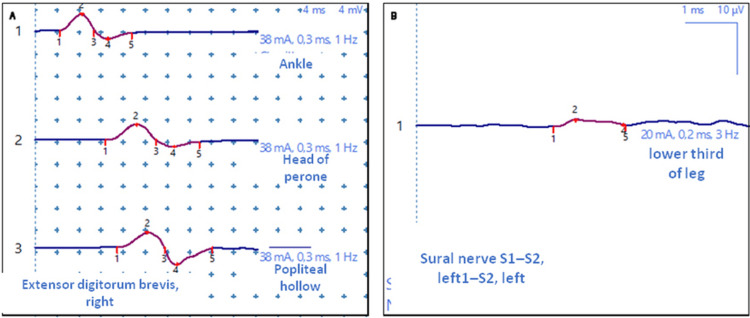
Representative motor and sensory nerve conduction traces (A) Motor responses of the common peroneal nerve recorded from the extensor digitorum brevis showed markedly reduced CMAP amplitudes without conduction block, consistent with axonal motor neuropathy. (B) The left sural SNAP demonstrated a low-amplitude sensory response, consistent with distal axonal sensory involvement. CMAP: compound muscle action potential, SNAP: sensory nerve action potential

Based on the clinical presentation, electrophysiological profile, profoundly low serum thiamine level, and systematic exclusion of alternative etiologies, a diagnosis of axonal sensorimotor polyneuropathy secondary to thiamine deficiency was established.

High-dose intravenous thiamine supplementation was promptly initiated at 500 mg three times daily for five days, followed by 500 mg once daily for ten days. Following completion of thiamine therapy, partial neurological improvement was observed; however, the patient remained wheelchair-dependent with marked impairment in activities of daily living. Formal functional assessment at admission to our PMR department revealed a Barthel index (BI) of 40/100, Berg balance scale (BBS) of 18/56 (high fall risk), functional independence measure (FIM) motor subscale of 28/91 (maximal assistance required), and MRC sum score of 42/60, warranting comprehensive inpatient rehabilitation.

A structured, multidisciplinary, and individualized inpatient rehabilitation program was subsequently implemented. Daily physiotherapy sessions (30 minutes per day, adjusted according to individual fatigue thresholds) focused on progressive resistance strengthening of proximal and distal muscle groups in all four limbs, trunk stabilization exercises, and progressive balance and postural control training in both sitting and standing positions. Gait retraining was initiated using parallel bars with body-weight support as required (Figure 3), then progressed to a walker with systematic fall-prevention strategies. This stepwise progression was associated with improved postural control and balance during ambulation, resulting in safer, more stable walking.

**Figure 3:**
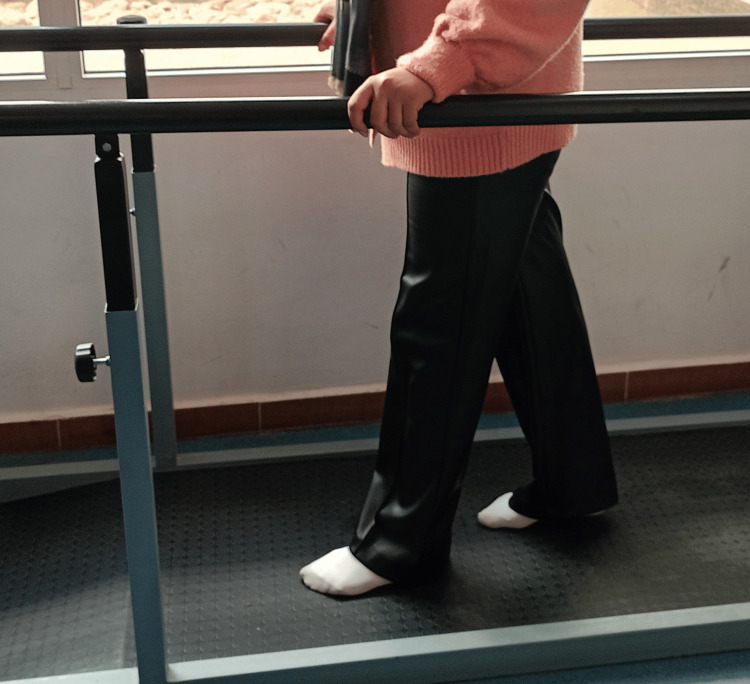
Supervised gait training within parallel bars during a rehabilitation session

Occupational therapy sessions aimed to restore independence in basic activities of daily living, introduce energy conservation techniques, and adapt functional tasks to pregnancy-related physical constraints. Regular rest breaks and continuous monitoring of maternal and fetal well-being were maintained throughout all sessions.

After four weeks of intensive inpatient rehabilitation, significant functional gains were documented: BI 75/100, BBS 42/56 (moderate fall risk), FIM motor subscale 68/91 (minimal to moderate assistance), and MRC sum score 52/60. The patient progressed from wheelchair dependence to ambulation with a walking frame. Ankle-foot orthoses were prescribed to manage persistent bilateral foot drop, facilitating further gait progression to cane-assisted walking.

At two-month follow-up, near-complete functional independence was achieved: BI 95/100, BBS 52/56 (low fall risk), FIM motor subscale 88/91 (modified independence), and MRC sum score 58/60. The patient walked independently without assistive devices, with marked improvement in gait stability, balance, and overall functional autonomy.

## Discussion

Thiamine deficiency is an underdiagnosed cause of peripheral neuropathy, particularly in settings where malnutrition and hyperemesis gravidarum may coexist [1]. Thiamine is essential for neuronal energy metabolism, and its depletion may disrupt metabolic pathways and contribute to axonal dysfunction, consistent with the axonal sensorimotor polyneuropathy observed in our patient [4-5]. Pregnancy is a period of increased metabolic demand, and prolonged vomiting with poor nutritional intake can rapidly exhaust thiamine stores, making pregnant women vulnerable to deficiency-related neurological complications [1-6].

Several reports have highlighted thiamine deficiency-related neuropathy and myeloneuropathy in association with hyperemesis gravidarum and severe nutritional depletion, often presenting with rapidly progressive weakness and gait disturbance [7]. In these cases, prompt recognition and high-dose thiamine supplementation are essential to prevent progression and potential maternal-fetal complications [1,6,7]. Our patient presented with a subacute axonal sensorimotor polyneuropathy in the context of prolonged hyperemesis and documented thiamine deficiency, with normal brain MRI, which is consistent with previously described non-alcoholic thiamine deficiency-related neuropathies [2-6].

Our patient demonstrated predominantly axonal sensorimotor polyneuropathy with markedly reduced CMAP and SNAP amplitudes (common peroneal CMAP 2.9 mV, sural SNAP 1.4 μV) but preserved conduction velocities (41.7-48.1 m/s), consistent with the electrophysiological signature reported in acute thiamine-deficiency neuropathy. Koike et al. described three patients with acute axonal polyneuropathy and Wernicke-Korsakoff syndrome who showed similar findings: severely decreased amplitudes with minimal conduction slowing, which improved dramatically within two weeks of intravenous thiamine. This pattern reflects physiological conduction failure rather than structural demyelination, explaining the rapid reversibility observed with thiamine replacement. In contrast, chronic alcoholic neuropathy often demonstrates mixed axonal-demyelinating features with more prominent conduction velocity slowing, suggesting distinct pathophysiological mechanisms. The absence of conduction block, normal F-wave latencies, and preserved motor responses in our patient's upper limbs further support a length-dependent axonal process rather than immune-mediated demyelination [8].

The differential diagnosis of acute-to-subacute polyneuropathy in pregnancy includes Guillain-Barré syndrome (GBS), chronic inflammatory demyelinating polyradiculoneuropathy (CIDP), other nutritional deficiencies (thiamine, copper, vitamin E), and toxic neuropathies. In our patient, GBS was initially considered given the ascending weakness and areflexia; however, cerebrospinal fluid showed no albuminocytologic dissociation (protein 0.35 g/L, WBC 2/mm³), and electrophysiological findings were axonal rather than demyelinating. CIDP typically follows a relapsing-remitting course with demyelinating nerve conduction patterns, neither of which was observed. Other nutritional deficiencies were excluded by normal serum thiamine (320 pg/mL) and folate (8.5 ng/mL) levels. The combination of documented severe hyperemesis gravidarum, profoundly low serum thiamine (9 µg/L), characteristic axonal pattern, and absence of alternative causes firmly established thiamine deficiency as the etiology [6-9].

Published recovery timelines for thiamine-deficiency neuropathy vary widely, ranging from two weeks to 12 months, with most neurological improvement occurring within the first two to three months of thiamine replacement. Our patient achieved independent ambulation within two months, consistent with favorable outcomes reported in early-treated cases without central nervous system involvement. Koike et al. reported symptom improvement within two weeks, with corresponding electrophysiological recovery, while other studies indicate that peripheral neuropathy recovers more slowly, typically requiring two to six months for significant improvement and up to 12 months for maximal recovery. Prognosis is significantly worse when diagnosis is delayed or complicated by Wernicke encephalopathy; in such cases, permanent neurological deficits occur in 19-49% of patients, and mortality may reach 20%. The normal brain MRI in our patient, absence of encephalopathic features, and prompt initiation of high-dose parenteral thiamine (500 mg three times daily) likely contributed to the excellent functional recovery observed [8-10].

Beyond etiologic treatment, supportive care and rehabilitation are critical components of management for peripheral neuropathies [11]. Rehabilitation programs can improve balance and functional performance in populations with peripheral neuropathy, and some interventions are associated with reduced fall risk [12].

While thiamine replacement addresses the underlying metabolic defect, structured rehabilitation is essential for motor relearning, balance retraining, and the development of compensation strategies. In our setting, a structured multidisciplinary rehabilitation program consisted of a coordinated, goal-oriented intervention provided by a team that included a physiatrist, physiotherapists, and occupational therapists. The program included weekly reassessments and individualized adjustments of treatment targets based on functional progress and pregnancy-related limitations. Objective functional data demonstrate the specific contribution of rehabilitation beyond nutritional therapy alone: at transfer to our department after 10 days of intravenous thiamine, the patient remained wheelchair-dependent with severe functional limitation (BI 40/100, BBS 18/56, FIM motor subscale 28/91).

After four weeks of intensive multidisciplinary rehabilitation, including progressive strengthening of proximal and distal muscles, balance and postural control training, gait re-education with parallel bars and assistive devices, and orthotic management of foot drop, functional scores improved dramatically: BI 75/100, BBS 42/56, and FIM motor 68/91, with progression to walker ambulation. By two months, she achieved near-complete independence (BI 95/100, BBS 52/56, FIM motor 88/91, MRC sum score 58/60) and walked independently without assistive devices. These sustained functional gains occurred well beyond the expected timeframe for neurological recovery from thiamine replacement alone (which typically plateaus within two to four weeks), strongly supporting the additive value of structured rehabilitation in optimizing functional outcomes in thiamine-deficiency neuropathy [13-14].

This case calls for heightened awareness among obstetricians, neurologists, and rehabilitation physicians regarding nutritional neuropathies in pregnant women with prolonged vomiting. Early screening for thiamine deficiency, prompt high-dose parenteral supplementation (≥100 mg daily prophylactically, 500-1500 mg daily for symptomatic patients), and timely referral to rehabilitation services may prevent permanent disability and optimize long-term functional outcomes [12].

## Conclusions

Peripheral neuropathy related to thiamine deficiency in pregnancy remains a rare and likely underrecognized condition, particularly in women with prolonged vomiting and poor nutritional intake. In such high-risk patients, early consideration of thiamine deficiency is essential to avoid delayed diagnosis and potentially irreversible disability.

This case highlights that a systematic diagnostic approach, combined with prompt parenteral thiamine replacement and timely multidisciplinary rehabilitation, was associated with substantial functional improvement, as reflected in validated outcome measures. However, given the inherent limitations of a single case report and the absence of comparative data, these observations should be interpreted with caution, and no causal conclusions can be drawn regarding the specific effect of rehabilitation. Clinicians should nonetheless maintain a low threshold for recognizing and treating thiamine deficiency in pregnant women with hyperemesis gravidarum and consider early referral to rehabilitation services to support functional recovery.
